# The Deactivation Mechanism of the Mo-Ce/Zr-PILC Catalyst Induced by Pb for the Selective Catalytic Reduction of NO with NH_3_

**DOI:** 10.3390/nano11102641

**Published:** 2021-10-07

**Authors:** Chenxi Li, Jin Cheng, Qing Ye, Fanwei Meng, Xinpeng Wang, Hongxing Dai

**Affiliations:** 1 Key Laboratory of Beijing on Regional Air Pollution Control, Department of Environmental Science, School of Environmental and Chemical Engineering, Faculty of Environment and Life, Beijing University of Technology, Beijing 100124, China; lcx@emails.bjut.edu.cn (C.L.); chengjin3708@163.com (J.C.); MengFanwei@emails.bjut.edu.cn (F.M.); 17121679@bjtu.edu.cn (X.W.); 2 Beijing Key Laboratory for Green Catalysis and Separation, Key Laboratory of Beijing on Regional Air Pollution Control, Key Laboratory of Advanced Functional Materials, Education Ministry of China, and Laboratory of Catalysis Chemistry and Nanoscience, Department of Environmental Chemical Engineering, School of Environmental and Chemical Engineering, Faculty of Environment and Life, Beijing University of Technology, Beijing 100124, China

**Keywords:** Pb poisoning, Mo-Ce/Zr-PILC catalyst, NH_3_-SCR, surface acidity

## Abstract

As a heavy metal, Pb is one component in coal-fired flue gas and is widely considered to have a strong negative effect on catalyst activity in the selective catalytic reduction of NO*_x_* by NH_3_ (NH_3_-SCR). In this paper, we investigated the deactivation mechanism of the Mo-Ce/Zr-PILC catalyst induced by Pb in detail. We found that NO conversion over the 3Mo4Ce/Zr-PILC catalyst decreased greatly after the addition of Pb. The more severe deactivation induced by Pb was attributed to low surface area, lower amounts of chemisorbed oxygen species and surface Ce^3+^, and lower redox ability and surface acidity (especially a low number of Brønsted acid sites). Furthermore, the addition of Pb inhibited the formation of highly active intermediate nitrate species generated on the surface of the catalyst, hence decreasing the NH_3_-SCR activity.

## 1. Introduction

Nitrogen oxides (NO*_x_*) are not only harmful to human health but are also important precursors of secondary pollution (e.g., photochemical smog, fine particles, and acid deposition) [1,2,3]. NH_3_-SCR is an efficient and economical technology that has recently been used in the elimination of NO*_x_* from the exhausts of stationary and mobile sources. In addition, the V_2_O_5_−WO_3_ (MoO_3_)/TiO_2_ catalysts are the most widely used catalyst systems in stationary sources [4]. However, these systems still have some inevitable practical defects, such as low N_2_ selectivity in the high-temperature range, high SO_2_ to SO_3_ conversion rates, and the strong biological toxicity of vanadium species [5]. Therefore, it is urgent and necessary to develop a new SCR catalyst with low toxicity and high activity. Among the possible substitutes, Ce-based oxides have attracted extensive attention due to their excellent oxygen storage/release ability and redox performance [6,7]. It is reported that Mo modification could significantly promote the adsorption and activation of NH_3_ species, which is beneficial for improvement in NH_3_-SCR activity [8]. Therefore, Mo-Ce mixed-oxide catalysts have been extensively investigated. It was found that these materials were the most promising SCR catalysts [9], among which the Mo-Ce/Zr-PILC catalysts exhibited outstanding NH_3_-SCR activity.

Deactivation induced by the poisoning of K, Na, Ca, Pb, or As is the main reason for the poor performance of the SCR catalysts. A number of experiments have been focused on the poisoning effects of alkali or alkaline earth metals on the NH_3_-SCR catalyst [10,11]. However, very few studies have been done regarding the effects of heavy metals on the activity of SCR catalysts. Pb is a typical heavy metal contained in the flue gas derived from coal-fired and municipal solid waste incineration power plants. Tokarz et al. [12] observed a strong accumulation effect of Pb on the NH_3_-SCR catalyst, where the Pb concentration reached 3350 ppm after 1908 h operation of a municipal waste incinerator. This finding revealed that lead dust exhibited a strong accumulative poisoning effect on the NH_3_-SCR catalyst. However, there have been few reports in the literature on the poisoning mechanisms of heavy metals (e.g., Pb) on NH_3_-SCR catalysts. Hence, it is necessary to investigate the effects of Pb compounds on the performance of NH_3_-SCR catalysts and the involved deactivation mechanisms.

To elucidate the inhibitory effect of Pb on Mo-Ce/Zr-PILC in the NH_3_-SCR reaction, we prepared 3Mo4Ce/Zr-PILC (Mo and Ce contents were 3 and 4 wt%, respectively) and Pb-doped 3Mo4Ce/Zr-PILC catalysts. The physicochemical properties of these samples were characterized using the BET, XRD, H_2_-TPR, XPS, NH_3_-TPD, and in situ DRIFTS techniques. The main purpose of the present work is to clarify the effect of Pb doping on the catalytic performance of Ce-based catalysts, and to elucidate the involved poisoning mechanisms.

## 2. Experimental

### 2.1. Catalyst Preparation

The zirconium pillared clay was prepared using the pillaring method. An appropriate amount of zirconium oxychloride solution was added dropwise to the montmorillonite suspension, stirred for 12 h, aged at ambient room conditions for 24 h, dried at 100 °C for 12 h, and calcined at 400 °C for 2 h (named as Zr-PILC). The 3Mo/Zr-PILC catalyst with a Mo loading of 3 wt% was prepared by impregnating Zr-PILC with a proper amount of ammonium molybdate under stirring for 1 h, and then was put into a rotary evaporator to dry and calcine in air at 400 °C for 2 h. The 3Mo4Ce/Zr-PILC (Ce content was 4 wt%) catalyst was prepared by impregnating the 3Mo/Zr-PILC powders with a Ce(NO_3_)_3_ aqueous solution, and its preparation method followed the steps above. The catalysts poisoned by Pb were prepared using the impregnating method, by mixing the 3Mo4Ce/Zr-PILC catalyst with Pb(CH_3_COO)_2_·3H_2_O at different molar Pb/Ce ratios, and the subsequent procedures were the same as the steps above. 

### 2.2. Catalyst Characterization

The crystal structures of the catalysts were determined using a Bruker D8 advance diffractometer (Bruker, Karlsruhe, Germany) equipped with a Cu K*α* detector. N_2_ adsorption−desorption isotherms were measured at −196 °C on a W-BK132F apparatus (JWGB, Beijing, China). X-ray photoelectron spectroscopy (XPS, ESCALAB 250Xi, Thermo Fisher, Waltham, MA, USA) with Al K*α* radiation (1486.6 eV) was used to analyze the surface compositions, metal oxidation states, and oxygen species of the catalysts. The sample powders were vacuumed, heated, and glued to the tape, and then the test was carried out immediately. The hydrogen temperature-programmed reduction (H_2_-TPR) and ammonia temperature-programmed desorption (NH_3_-TPD) experiments were carried out in a quartz U-tube reactor on a PCA-1200 analyzer (Beijing Builder Electronic Technology, Beijing, China) equipped with a thermal conductivity detector (TCD). Before H_2_-TPR measurement, the catalyst was pretreated in 5 vol% O_2_/N_2_ flow at 400 °C for 1 h and then cooled to room temperature (RT). The H_2_-TPR profiles were recorded in a 5 vol% H_2_/N_2_ flow of 30 mL/min from RT to 950 °C at a heating rate of 10 °C/min. Prior to NH_3_-TPD measurement, the catalyst was pretreated in a He flow of 30 mL/min at 400 °C for 1 h, followed by NH_3_ adsorption at 100 °C. Subsequently, the catalyst was purged in a He flow of 30 mL/min for 30 min to remove the physically adsorbed NH_3_. Finally, the NH_3_-TPD profiles were recorded in a He flow of 30 mL/min from 30 to 850 °C at a heating rate of 10 °C/min. The in situ diffuse reflectance Fourier transform infrared spectroscopy (in situ DRIFTS) experiments were performed on a Bruker TENSOR II spectrometer (Bruker, Karlsruhe, Germany). The catalyst was pretreated at 400 °C for 1 h to remove any adsorbed moisture and impurities. The background spectra were recorded by exposing the catalysts to a N_2_ flow of 100 mL/min at 200 °C. The absorption spectra of NH_3_ and (NO + O_2_) were recorded in a 100 mL/min flow of (1100 ppm NH_3_ + 1000 ppm NO + 4 vol% O_2_ + N_2_ (balance) and (1000 ppm NO + 4 vol% O_2_ + N_2_ (balance)), respectively.

### 2.3. NH_3_-SCR Activity Evaluation

The activity measurements were performed in a fixed-bed quartz reactor using 0.3 g of the catalyst (40–60 mesh) mixed with 0.3 g of quartz sand (40–60 mesh). The simulated exhaust gas was composed of 1100 ppm NH_3_ + 1000 ppm NO + 4 vol% O_2_ + 5 vol% H_2_O + N_2_ (balance). The total flow rate of the feed gas was 500 mL/min, and the space velocity (SV) was 100,000 mL/(g h). NO concentrations were measured using a MODEL1080 analyser (Beijing SDL Technology, Beijing, China). The NO conversion was calculated according to the following equation:

$$\mathrm{NO}\;\mathrm{conversion}\left(\%\right)=\frac{{\left[\mathrm{NO}\right]}_{\mathrm{in}}-{\left[\mathrm{NO}\right]}_{\mathrm{out}}}{{\left[\mathrm{NO}\right]}_{\mathrm{in}}}\times 100\%$$

## 3. Results and Discussion

### 3.1. NH_3_-SCR Activity

**Figure 1:**
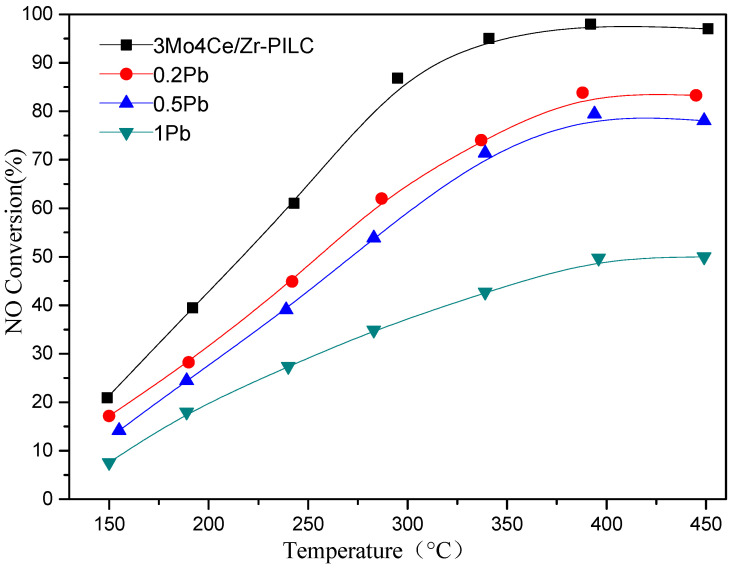
Catalytic activity for the NH_3_-SCR reaction over the 3Mo4Ce/Zr-PILC and Pb-poisoned 3Mo4Ce/Zr-PILC catalysts under the reaction conditions of (1000 ppm NO + 1100 ppm NH_3_ + 4 vol% O_2_ + 5 vol% H_2_O + N_2_ (balance)) and SV = 100,000 mL/(g h).

In the present study, we investigated the poisoning effects that Pb had on the catalytic performance of 3Mo4Ce/Zr-PILC. Figure 1 shows the NO conversion as a function of temperature (150–450 °C) over the 3Mo4Ce/Zr-PILC and Pb-poisoned 3Mo4Ce/Zr-PILC catalysts. Obviously, 3Mo4Ce/Zr-PILC exhibited excellent catalytic activity, with a NO conversion of 90% in the 300–450 °C range. However, with a rise in the doped Pb amount of 3Mo4Ce/Zr-PILC, NO conversion decreased considerably. When the Pb/Ce molar ratio was 0.2, catalytic efficiency in the whole temperature range was decreased by 5–25%. When the Pb/Ce molar ratio rose to 1, the activity of the catalyst was less than 50% at 150–450 °C. Obviously, the deNO*_x_* performance of the catalyst was greatly inhibited after Pb doping, and this inhibitive effect was enhanced with increasing Pb/Ce molar ratio.

### 3.2. Crystal Phase and Textural Characteristics

**Figure 2:**
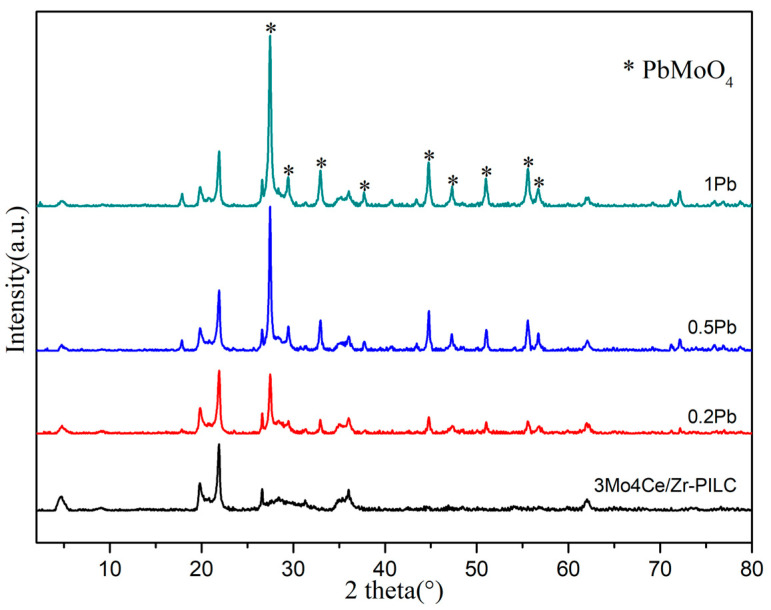
XRD patterns of the 3Mo4Ce/Zr-PILC and Pb-poisoned 3Mo4Ce/Zr-PILC catalysts.

The crystal phase compositions of the 3Mo4Ce/Zr-PILC and Pb-poisoned 3Mo4Ce/Zr-PILC catalysts were measured using XRD. Figure 2 shows their diffraction patterns. According to our previous research [13], the XRD pattern of the PILC sample displayed a high-intensity basement (001) reflection peak at 2*θ* = 9.3°, indicating that the montmorillonite clay possessed a regular and ordered interlayer structure. The (001) reflection peak of the 3Mo4Ce/Zr-PILC and Pb-poisoned 3Mo4Ce/Zr-PILC catalysts was shifted to a lower angle and its intensity decreased, indicating an increase in interlayer distance and a disorder in the structure of the clay, which was caused by the effect of the strong delamination when the metal oxide entered the interlayer of clay. For the 3Mo4Ce/Zr-PILC catalyst, two-dimensional *hk* reflections were present at 2*θ* = 19.8° and 34.9°, in which the former was a summation of the *hk* indices of (02) and (11), while the latter was a summation of the *hk* indices of (13) and (20) [14]. The peaks at 2*θ* = 26.6° and 28° were reflections of the quartz and cristobalite impurities in the clay [15]. No diffraction peaks assignable to the crystalline cubic CeO_2_ and MoO_3_ were detected, suggesting that the cerium and molybdenum species were widely dispersed on the Zr-PILC surface, or were present in an amorphous state. As shown in Figure 2, after Pb doping, some obvious diffraction signals assignable to the PbMoO_4_ phase were recorded at 2*θ* = 27.5°, 29.5°, 32.9°, 37.8°, 44.9°, 47.3°, 50.9°, 55.7°, and 56.7°, and the intensity of the diffraction peaks ascribable to the PbMoO_4_ phase increased gradually with a rise in Pb doping, indicating partial accumulation of the PbMoO_4_ phase. The accumulation of the PbMoO_4_ species would result in the partial deactivation of the 3Mo4Ce/Zr-PILC catalyst, which was consistent with its NH_3_-SCR activity.

**Figure 3:**
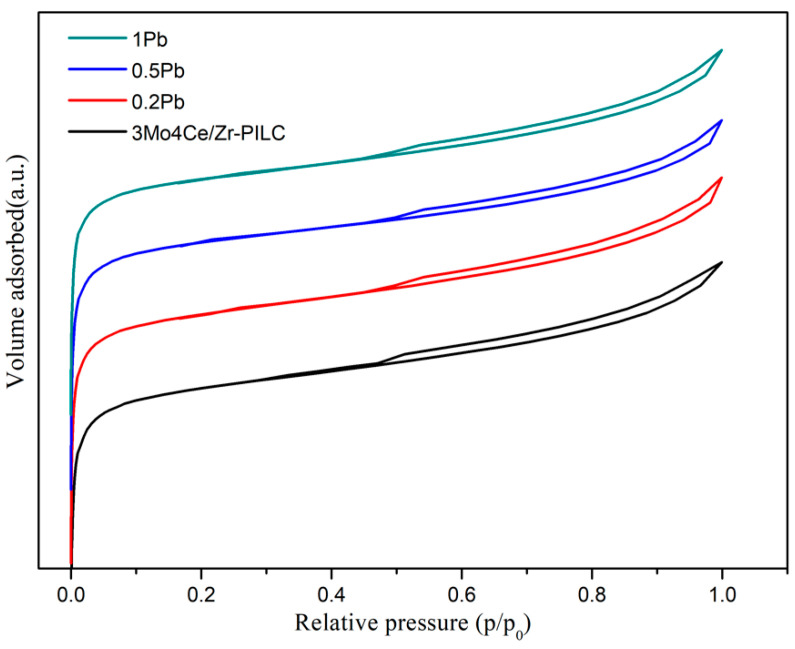
N_2_ adsorption-desorption isotherms of the 3Mo4Ce/Zr-PILC and Pb-poisoned 3Mo4Ce/Zr-PILC catalysts.

N_2_ adsorption–desorption isotherms of the 3Mo4Ce/Zr-PILC and Pb-poisoned 3Mo4Ce/Zr-PILC catalysts are shown in Figure 3. It can be seen that the isotherms of the catalysts were obviously in accordance with type I in the low relative pressure range, according to the IUPAC classification, which conformed to the characteristics of microporous materials [16]. The adsorbed volumes of the catalysts increased significantly in the *p*/*p*_0_ (<0.01) range, which was due to the formation of a large number of micropores during the pillaring process. Additionally, the hysteresis loops of all of the catalysts corresponded to type H3, which appeared in the higher relative pressure range, indicating the presence of mesopores.

The textural parameters of the 3Mo4Ce/Zr-PILC and Pb-poisoned catalysts are listed in Table 1. As seen in Table 1, 3Mo4Ce/Zr-PILC displayed a surface area and a pore volume of 270 m^2^/g and 0.164 cm^3^/g, respectively. Additionally, it was noticeable that the doping of Pb resulted in significant decreases in surface area and pore volume, which might be attributable to the partial blocking of the micropores in the catalysts, the agglomeration of the particles, or both.

**Figure 4:**
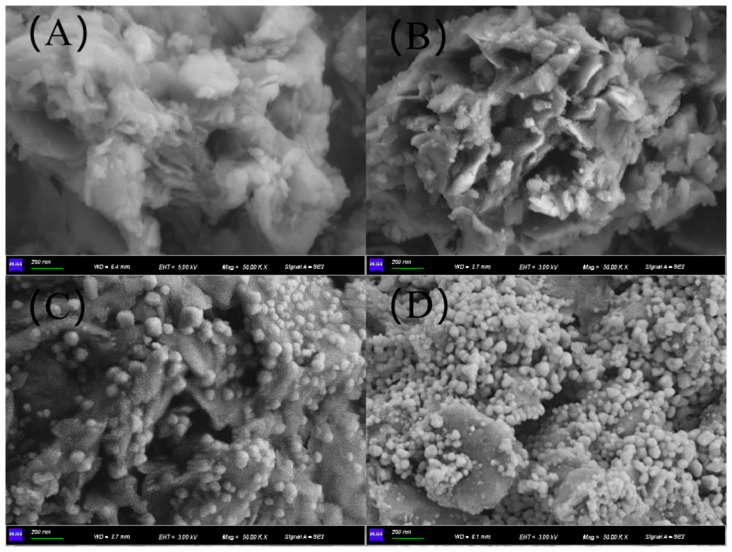
SEM images of (**A**) 3Mo4Ce/Zr-PILC, (**B**) 0.2Pb-Mo4Ce/Zr-PILC, (**C**) 0.5Pb-Mo4Ce/Zr-PILC, and (**D**) 1Pb-Mo4Ce/Zr-PILC.

The SEM images of the 3Mo4Ce/Zr-PILC and Pb-poisoned 3Mo4Ce/Zr-PILC catalysts are shown in Figure 4. It can be observed that the 3Mo4Ce/Zr-PILC catalyst displayed a microporous structure, while particles appeared on the surface of the Pb-poisoned catalysts. Moreover, for the Pb-poisoned 3Mo4Ce/Zr-PILC catalysts with higher quantities of doped Pb, the number of particles increased but the pore size decreased, which was consistent with the BET results.

### 3.3. Redox Properties

**Figure 5:**
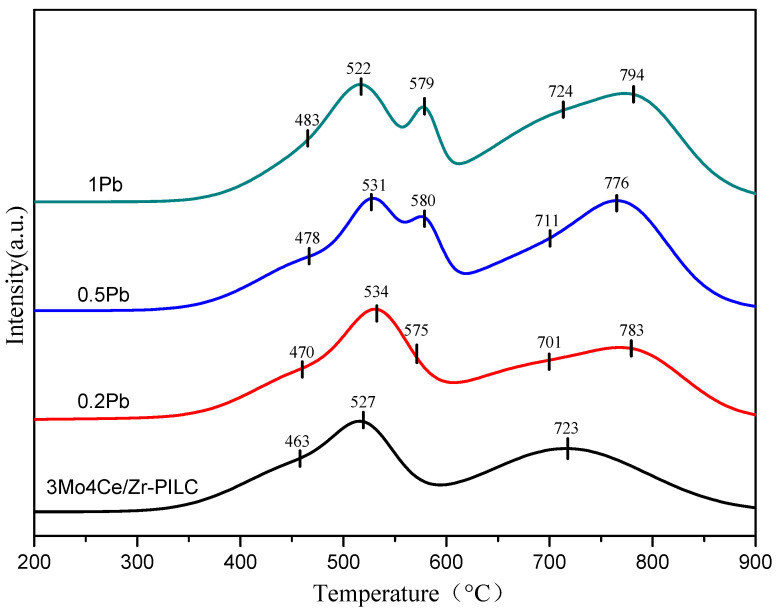
H_2_-TPR profiles of the 3Mo4Ce/Zr-PILC and Pb-poisoned 3Mo4Ce/Zr-PILC catalysts.

The redox property of a catalyst is an important factor in NH_3_-SCR reactions. The reducibility of Ce and Mo species in Pb-free and Pb-doped catalysts was investigated using the H_2_-TPR technique, so that the effect of Pb doping on the catalytic activity of 3Mo4Ce/Zr-PILC could be clarified. Meanwhile, after quantitative analysis of the H_2_-TPR peaks, the H_2_ consumption and reduction peak positions are summarized in Table 2. H_2_-TPR profile of 3Mo4Ce/Zr-PILC displayed three main reduction peaks at 463, 527, and 723 °C (Figure 5), which could be interpreted as the reduction of the surface Ce^4+^ to Ce^3+^ species, co-reduction of the octahedrally structured well-dispersed Mo and iron species, and the co-reduction of the tetrahedrally structured Mo species and bulk CeO_2_ species [17,18,19], respectively. In profiles of the Pb-poisoned catalysts, the first peak due to the reduction of the Ce species was shifted to a higher temperature, indicating that doping of Pb stabilized the Ce species and made it less reducible. The temperature of the low-temperature reduction peak increased in the order 3Mo4Ce/Zr-PILC (463 °C) > 0.2Pb-3Mo4Ce/Zr-PILC (470 °C) > 0.5Pb-3Mo4Ce/Zr-PILC (478 °C) > 1Pb-3Mo4Ce/Zr-PILC (483 °C), which was in accordance with their changing trend in SCR performance. In addition, two new reduction peaks at 575 and 790 °C appeared in the Pb-poisoned catalysts, which might be attributable to the reduction of the surface or bulk oxygen coordinated with the Pb atoms [20,21].

### 3.4. Surface Property

**Figure 6:**
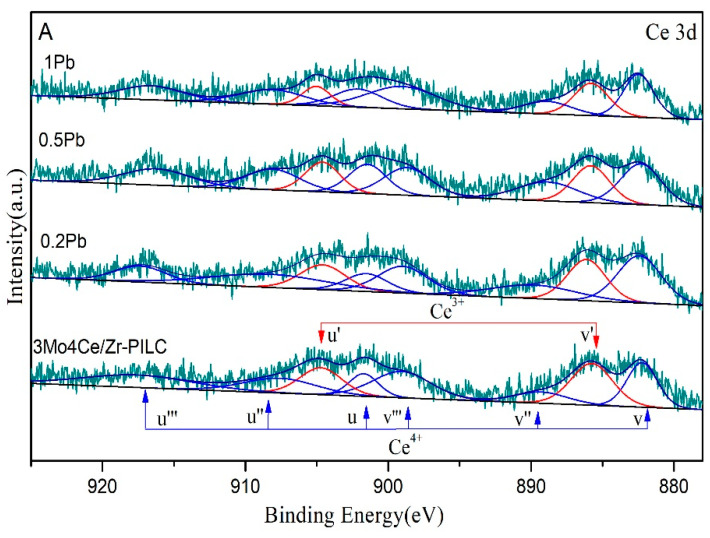

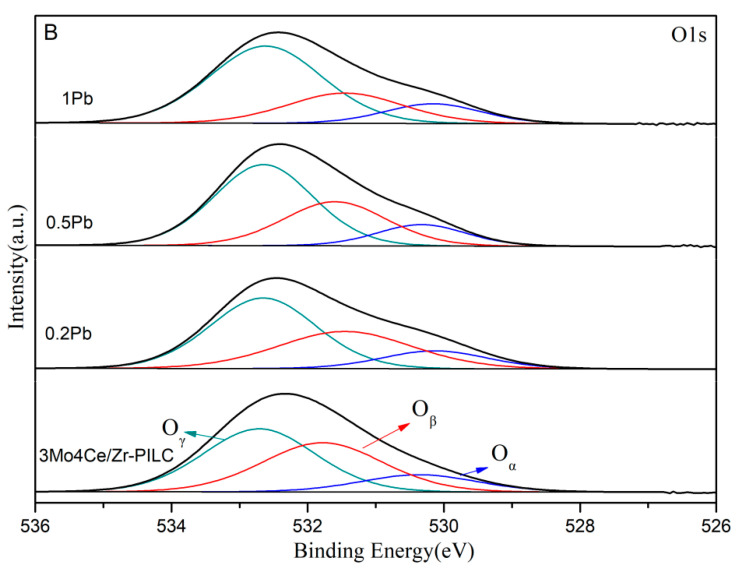
(**A**) Ce 3d and (**B**) O 1s spectra of the 3Mo4Ce/Zr-PILC and Pb-poisoned 3Mo4Ce/Zr-PILC catalysts.

To gain better insights into the surface compositions and chemical states of the catalysts, we conducted XPS characterization, and the results are shown in Figure 6 and Table 3.

The Ce 3d XPS spectra of the 3Mo4Ce/Zr-PILC and Pb-poisoned 3Mo4Ce/Zr-PILC catalysts are presented in Figure 6A. The peaks labeled as *v* and *u* corresponded to Ce 3d_5/2_ and Ce 3d_3/2_ spin-orbit peaks [22,23], respectively, which could be deconvoluted into eight components: the *u^I^* and *v^I^* components were assigned to the surface Ce^3+^ species corresponding to the 3*d*^10^4*f*^1^ initial electronic state, while the other components were due to the surface Ce^4+^ species corresponding to the 3*d*^10^4*f*^0^ initial electronic state. The Ce^3+^/Ce^4+^ atomic ratios were calculated according to the integral areas of the corresponding peaks. With an increase in Pb doping, the Ce^3+^/Ce^4+^ atomic ratio decreased in the order 3Mo4Ce/Zr-PILC (0.43) > 0.2Pb-3Mo4Ce/Zr-PILC (0.36) > 0.5Pb-3Mo4Ce/Zr-PILC (0.32) > 1Pb-3Mo4Ce/Zr-PILC (0.27), which was the same trend seen in the catalytic activity. The Ce^3+^ species exerted a positive effect on catalytic activity owing to its strong ability to create charge imbalance and form oxygen vacancies and unsaturated chemical bonds on the catalyst surface, which could increase the amount of surface chemisorbed oxygen species [24]. Some oxygen vacancies were occupied due to Pb introduction, which inhibited the transformation of Ce^4+^ to Ce^3+^. Therefore, the decrease in the amount of the surface Ce^3+^ species on 1Pb-3Mo4Ce/Zr-PILC was an important parameter that gave rise to this system’s worse catalytic activity.

O 1s XPS spectra of the catalysts are illustrated in Figure 6B, which could be decomposed into three components at BE = 532.61–532.70, 531.48–531.78, and 530.16–531.36 eV, assignable to the surface lattice oxygen of the Si–O bond (O*_γ_*), chemisorbed oxygen (O*_β_*), and lattice oxygen (O*_α_*) [25,26,27], respectively. Chemisorbed oxygen was mainly the oxygen species adsorbed at the oxygen vacancies, as well as the defect oxides and hydroxyl-like groups. Based on that curve-fitting approach, the O*_β_* concentrations on the surface of the catalysts were calculated, and the results are listed in Table 3. A distinct decrease in the amount of O*_β_* species was observed after the Pb doping. The O*_β_*/(O*_α_* + O*_β_* + O*_γ_*) molar ratio decreased in the order 3Mo4Ce/Zr-PILC (0.394) > 0.2Pb-3Mo4Ce/Zr-PILC (0.347) > 0.5Pb-3Mo4Ce/Zr-PILC (0.314) > 1Pb-3Mo4Ce/Zr-PILC (0.248). These results were consistent with the order of NH_3_-SCR activity, indicating that the lower amounts of O*_β_* species on the Pb-poisoned catalysts were also responsible for their lower catalytic activities. It has been previously demonstrated that O*_β_* species are the most effective oxygen species in the SCR reaction [27]. Moreover, the oxygen in the gas phase and the oxygen adsorbed on the catalyst surface could be easily exchanged with the O*_β_* species, and the active transportation of the O*_β_* species could also facilitate the SCR reaction [28].

### 3.5. Surface Acidity

**Figure 7:**
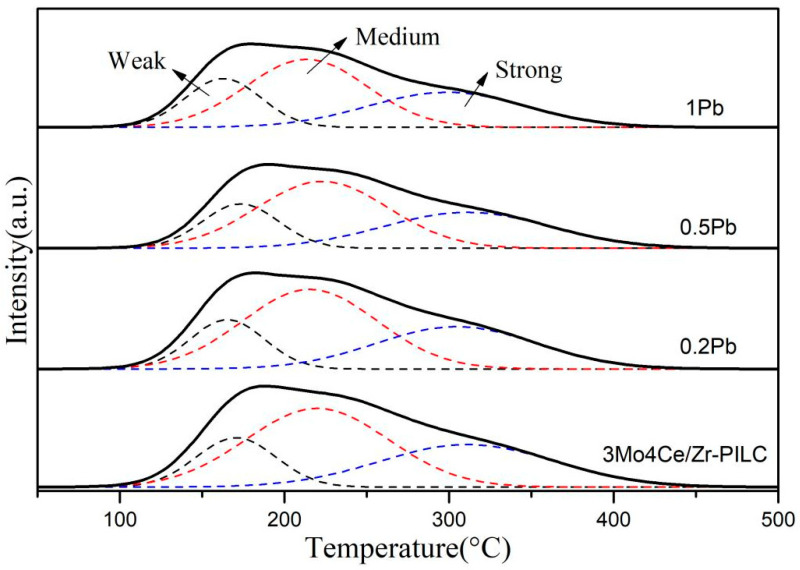
NH_3_-TPD profiles of the 3Mo4Ce/Zr-PILC and Pb-poisoned 3Mo4Ce/Zr-PILC catalysts.

The NH_3_ adsorption capacity has been widely reported to influence the SCR reaction, and it has also been strongly associated with the surface acidity of a catalyst [29]. Therefore, NH_3_-TPD experiments on the 3Mo4Ce/Zr-PILC and Pb-poisoned 3Mo4Ce/Zr-PILC catalysts were carried out to examine the influence of Pb doping on surface acidity, and the results are illustrated in Figure 7.

There were three desorption peaks in the NH_3_-TPD profile of each sample (Figure 7). The first peak was located at 163–173 °C, and signified the desorption of the physically adsorbed NH_3_ species and some NH_4_^+^ species at the weak Brønsted acid sites (i.e., weak acid sites); the second peak at 214–223 °C was attributed to the NH_4_^+^ species desorbed from the strong Brønsted acid sites (i.e., medium acid sites); and the third peak at 301–311 °C was assigned to the desorption of the coordinated NH_3_ bound to the Lewis acid sites (i.e., strong acid sites) [29,30]. Acid site numbers were quantitatively analyzed according to the NH_3_-TPD profiles, as listed in Table 4. The amount of Brønsted acid sites was in the order: 3Mo4Ce/Zr-PILC (0.131 mmol/g) > 0.2Pb-3Mo4Ce/Zr-PILC (0.125 mmol/g) > 0.5Pb-3Mo4Ce/Zr-PILC (0.103 mmol/g) > 1Pb-3Mo4Ce/Zr-PILC (0.096 mmol/g). The total acid amount decreased in the sequence 3Mo4Ce/Zr-PILC (0.264 mmol/g) > 0.2Pb-3Mo4Ce/Zr-PILC (0.247 mmol/g) > 0.5Pb-3Mo4Ce/Zr-PILC (0.213 mmol/g) > 1Pb-3Mo4Ce/Zr-PILC (0.202 mmol/g). It is noteworthy that a substantial decrease in the desorbed ammonia amount was observed when the amount of doped Pb increased. One reason for this phenomenon was probably due to the agglomeration of Pb species on the catalyst surface, partially blocking the micropores and covering some of the acid sites in the catalyst. In addition, it can be seen from the above Ce 3d XPS results that the concentration of the surface Ce^3+^ species on the sample decreased after Pb doping. According to the literature [31], the decrease in Ce^3+^ atomic concentration leads to a decrease in amount of oxide defects or hydroxyl-like groups, resulting in a decrease in the number of Brønsted acid sites. Therefore, we can conclude that the reduced number of acid sites (especially Brønsted acid sites) in the Pb-poisoned catalysts was one of the main reasons for the decrease in catalytic activity.

### 3.6. Switching Feed Gas from NO + O_2_ + N_2_ + NH_3_ to NO + O_2_ + N_2_ on Various Catalysts

**Figure 8:**
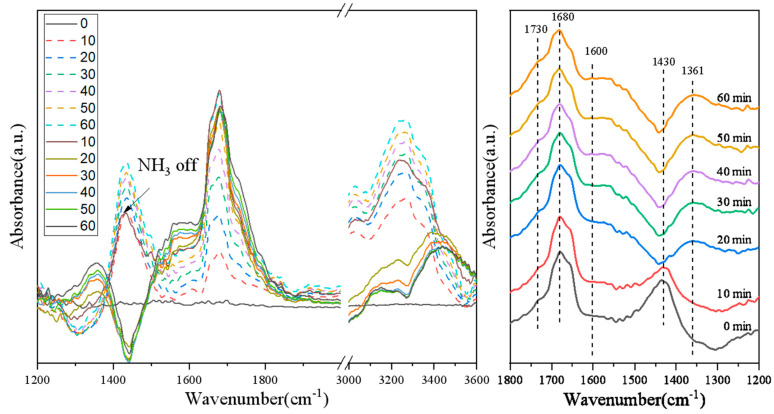
In situ DRIFTS spectra of the 3Mo4Ce/Zr-PILC catalyst exposed to (1100 ppm NH_3_ + 1000 ppm NO + 4% O_2_ + N_2_ (balance)) for 1 h and then switched to (1000 ppm NO + 4% O_2_ + N_2_ (balance)) for 1 h at 200 °C.

In order to investigate activity of the NO*_x_* species and their role in the NH_3_-SCR reaction, transient experiments were performed over the 3Mo4Ce/Zr-PILC catalyst. Figure 8 shows the DRIFTS spectra at 200 °C recorded after switching from 1100 ppm NH_3_ + 1000 ppm NO + 4% O_2_ + N_2_ (balance) to a flow containing 1000 ppm NO + 4% O_2_ + N_2_ (balance) over the 3Mo4Ce/Zr-PILC catalyst.

From the transient experimental results of the 3Mo4Ce/Zr-PILC catalyst, it can be seen that bands attributable to the nitrate species and NH_3_ species appeared on the surface of 3Mo4Ce/Zr-PILC after the flow containing NH_3_ + NO + O_2_ + N_2_ was introduced. Several bands were observed at 1430, 1600, 1680, and 3200–3400 cm^−1^, in which the bands at 1430 and 1680 cm^–1^ were attributed to the symmetric bending vibrations of NH_4_^+^ that was chemisorbed at the Brønsted acid sites, and the band at 3200–3400 cm^–1^ was assigned to the NH_3_ species adsorbed at the Lewis acid sites [32]. In addition, the band at 1600 cm^–1^ could be ascribed to the bridged nitrate, and the band attributable to the adsorbed nitrogen oxide species was at 1680 cm^–1^ [31,33]. It is well known that the adsorbed NO_2_ species can participate in the “fast NH_3_-SCR” reaction, which promotes the NH_3_-SCR performance [34]. When the feed gas was switched to NO + O_2_ + N_2_, the bands at 1430 and 3200–3400 cm^–1^ disappeared rapidly with respect to time, indicating that the NH_3_ adsorbed at the Lewis and Brønsted acid sites reacted with the nitrate species, and the NH_3_ species were gradually consumed during the reaction. Simultaneously, the intensity of the bands at 1331 and 1600 cm^−1^ was attributable to monodentate nitrate, and bridge nitrate also increased rapidly, indicating that the monodentate nitrates and bridge nitrates were highly active NO*_x_* species and played an important role in the NH_3_-SCR reaction.

**Figure 9:**
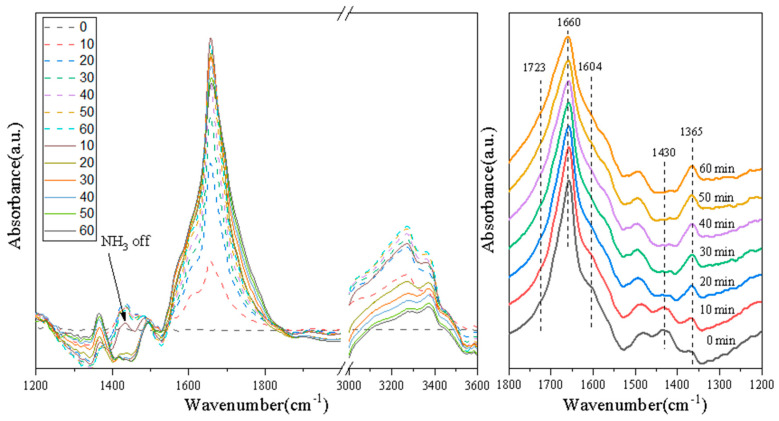
In situ DRIFTS spectra of the 1Pb-3Mo4Ce/Zr-PILC catalyst exposed to (1100 ppm NH_3_ + 1000 ppm NO + 4% O_2_ + N_2_ (balance)) for 1 h and then switched to (1000 ppm NO + 4% O_2_ + N_2_ (balance)) for 1 h at 200 °C.

Figure 9 shows the DRIFTS spectra of the 1Pb-3Mo4Ce/Zr-PILC catalyst. The band intensity of the NH_3_ species at 1430 and 3200–3400 cm^−1^ was lower than that of the 3Mo4Ce/Zr-PILC catalyst after exposure to a flow of NH_3_ + NO + O_2_ + N_2_. The intensity of the bands at 1365 and 1604 cm^−1^ was attributable to the monodentate nitrate, and bridge nitrate also increased slightly after cutting off NH_3_ introduction. These results indicate that the doping of Pb inhibited the adsorption of the NH_3_ species on the 1Pb-3Mo4Ce/Zr-PILC catalyst, and less of the main active species monodentate nitrate and bridge nitrate were generated, which would influence NH_3_-SCR activity of the catalyst.

Therefore, it can be concluded that the monodentate nitrate, bridge nitrate, and adsorbed NO_2_ species were the highly active intermediate species which could promote the NH_3_-SCR reaction. Meanwhile, the doping of Pb inhibited the formation of intermediate nitrate species on the surface of the catalyst, thereby inhibiting the NH_3_-SCR activity.

### 3.7. Pb Poisoning Mechanism

Pb is an ingredient found in coal-fired flue gas and inhibits SCR catalysts. The DeNO*_x_* performance of the catalyst was greatly suppressed after the Pb doping, and this inhibition effect was enhanced with increasing Pb/Ce molar ratio. XRD patterns (Figure 2) and SEM images (Figure 4) demonstrate that there was obvious agglomeration of the metal oxides after the Pb doping, and some PbMoO_4_ species were generated on the surface of the catalysts. In combination with the Ce 3d XPS results (Figure 6A), this suggests that Pb might occupy the oxygen vacancies in ceria, which could weaken the Ce^3+^ ↔ Ce^4+^ redox cycle and suppress the surface Ce^4+^ → Ce^3+^ transformation. In addition, based on the characterization results of NH_3_-TPD (Figure 7), the number of both Brønsted and Lewis acid sites decreased significantly (especially the number of Brønsted acid sites) after the Pb doping. Since NH_3_ was more readily adsorbed on the catalyst surface than NO in the SCR reaction, the adsorbed ammonia species were critical in the SCR reaction [35]. Furthermore, Pb inhibited the formation of the intermediate nitrate species (i.e., monodentate nitrates and bridge nitrates) on the surface of catalysts in the SCR reaction. The decrease in ammonia absorbance at the acid sites, the decrease of the highly active intermediate species, and the vanishing of active ammonia species were the most important influencing factors in the Pb-induced deactivation of the 3Mo4Ce/Zr-PILC catalyst.

## 4. Conclusions

Pb doping caused a serious deactivation of the 3Mo4Ce/Zr-PILC catalyst for the NH_3_-SCR reaction in this study. NO conversion decreased from 98% to 49% over the 3Mo4Ce/Zr-PILC catalyst at 450 °C when the Pb/Ce molar ratio was 1. The characterization results revealed that as the amount of surface Ce^3+^ and chemisorbed oxygen species increased, the interaction between PbO and 3Mo4Ce/Zr-PILC increased as well, with more PbMoO_4_ species formed on the catalyst surface, less intermediate nitrate species, and lower redox ability and surface acidity.

## Figures and Tables

**Table 1:** BET surface areas and pore volumes of the 3Mo4Ce/Zr-PILC and Pb-poisoned 3Mo4Ce/Zr-PILC catalysts.

Catalyst	BET Surface Area (m^2^/g)	Pore Volume (cm^3^/g)
3Mo4Ce/Zr-PILC	270	0.164
0.2Pb	259	0.161
0.5Pb	256	0.154
1Pb	243	0.152

**Table 2:** Reduction peak temperatures and H_2_ consumption of the 3Mo4Ce/Zr-PILC and Pb-poisoned 3Mo4Ce/Zr-PILC catalysts.

Catalyst		Reduction Peak Temperature (°C)				H_2_ Consumption (mmol/g)
	Peak 1	Peak 2	Peak 3	Peak 4	Peak 5	
3Mo4Ce/Zr-PILC	463	527	-	723	-	0.79
0.2Pb	470	534	575	701	783	0.90
0.5Pb	478	531	580	711	776	1.01
1Pb	483	522	579	724	794	1.10

**Table 3:** Surface element compositions of the 3Mo4Ce/Zr-PILC and Pb-poisoned 3Mo4Ce/Zr-PILC catalysts.

Catalyst	Composition of Cerium Species (at%)			Composition of Oxygen Species (mol%)		
	Ce^3+^	Ce^4+^	Ce^3+^/Ce^4+^ Atomic Ratio	O*_α_*	O*_β_*	O*_γ_*
3Mo4Ce/Zr-PILC	30.3	69.7	0.43	13.4	39.4	47.2
0.2Pb	26.2	73.8	0.36	13.1	34.7	52.2
0.5Pb	24.3	75.7	0.32	13.1	31.4	55.5
1Pb	21.1	78.9	0.27	13.4	24.8	61.8

**Table 4:** NH_3_-TPD temperatures and desorption amounts of the 3Mo4Ce/Zr-PILC and Pb-poisoned 3Mo4Ce/Zr-PILC catalysts.

Catalyst	Temperature (°C)			Acidity (mmol_NH3_/g)			Total Desorption Amount (mmol_NH3_/g) (mmol/g)
	Weak Peak	Medium Peak	Strong Peak	Weak Peak	Medium Peak	Strong Peak	
3Mo4Ce/Zr-PILC	171	220	311	0.046	0.131	0.087	0.264
0.2Pb	166	215	305	0.043	0.125	0.079	0.247
0.5Pb	173	223	310	0.040	0.103	0.070	0.213
1Pb	163	214	301	0.042	0.096	0.064	0.202

## Data Availability

The data presented in this study are available on request from the corresponding author.
